# Preliminary evaluation of a novel serotype O foot-and-mouth disease mRNA vaccine

**DOI:** 10.3389/fmicb.2025.1503191

**Published:** 2025-04-28

**Authors:** Jingang Zhao, Peng Xiao, Aiguo Xin, Heran Zhu, Hao Wang, Jinlong Xiao, Hong Gao

**Affiliations:** ^1^College of Animal Science and Technology, Yunnan Agricultural University, Kunming, China; ^2^Yunnan Tropical and Subtropical Animal Virus Disease Laboratory, Yunnan Academy of Animal Husbandry and Veterinary Sciences, Kunming, China; ^3^College of Veterinary Medicine, Yunnan Agricultural University, Kunming, China; ^4^National Foot-and-Mouth Disease Para-Reference Laboratory (Kunming), Yunnan Academy of Animal Husbandry and Veterinary Sciences, Kunming, China

**Keywords:** FMD, mRNA vaccine, immune protection, guinea pig, pig

## Abstract

Foot-and-mouth disease virus (FMDV) is one of the most significant animal pathogens worldwide, severely impacting the health and productivity of pigs, cattle, sheep, and other ungulates. Although the traditional vaccines have played a crucial role in epidemic control, inactivated vaccines face persistent challenges concerning the potential for virus dissemination and pressures from serotype and subtype matching. However, the manufacture of attenuated vaccines is forbidden, and the efficiency of alternative vaccines for immune protection is still inadequate. Consequently, there exists an urgent need for safer and more effective innovative vaccines in animal husbandry. In this study, we aimed to develop a lipid nanoparticle mRNA vaccine based on VP1-3A-3D epitopes from serotype O FMD and to verify its specific expression within cytoplasmic and injection sites. Our findings demonstrated that mRNA transfected into primary spleen cells derived from guinea pigs induced cytokine release, promoted differentiation of both CD4^+^ T and CD8^+^ T lymphocytes, and enhanced lymphocyte proliferation rates. Following immunization of mRNA vaccine in guinea pigs, we observed increased differentiation of both CD4^+^ T and CD8^+^ T cells, alongside elevated levels of cytokine secretion. Additionally, this vaccination induced the production of specific IgG antibodies as well as neutralizing antibodies. Importantly, our vaccine provided complete protection for all six guinea pigs against a lethal challenge of 100 GPID_50_, with histopathological scores indicating protection equivalent to that conferred by the inactivated vaccine. The viral load results demonstrated that the vaccine group significantly reduced viral copy numbers in serum and effectively decreased the concentration of the inflammatory cytokine IL-1β. Furthermore, during the pre-immune phase following vaccination with the mRNA vaccine in pigs, heightened cytokine secretion was observed, along with the inhibition of viral replication. Simultaneously, the neutralizing antibody titer in the serum remained stable over 4 months. Immunofluorescence analysis of spleen tissues from both guinea pigs and pigs demonstrated marked activation and increased expression of CD4^+^ and CD8^+^ T lymphocytes, as well as macrophages, in the mRNA vaccine group. In summary, this study suggests that the serotype O FMD mRNA vaccine is a promising candidate for further development in the fight against FMDV.

## 1 Introduction

Foot-and-mouth disease (FMD) is elicited by the FMD virus (FMDV) in the genus Aphthovirus of the family Picornaviridae (Zell et al., 2017). FMD is considered one of the most severe animal diseases globally, with the potential to induce acute febrile infectious diseases that are easily transmissible by pigs, cattle, sheep and other wild cloven-hoofed animals (Mason et al., 2003; Klein, 2009). Outbreaks of FMD can lead to substantial economic losses and adversely impact regional commerce (Knight-Jones and Rushton, 2013). FMDV comprises seven serotypes (A, O, C, Asia1, SAT1, SAT2, and SAT3), each of which is further classified into several subtypes. Cross-immunity among these serotypes is inadequate, and protection among some strains of the same serotype is incomplete (Gubbins et al., 2022). The diversity and polymorphism of the FMDV significantly complicate its prevention and management (Grubman and Baxt, 2004). Serotype O FMDV is one of the globally prevalent serotypes, persisting in many regions of Asia, and has been a major cause of recent FMD outbreaks in China (Ma et al., 2017; Li F. et al., 2023). Therefore, this study focuses on serotype O FMDV as the primary subject of investigation.

Over the past decade, the traditional inactivated vaccine has proven to be an effective tool in controlling and, in some cases, eliminating FMD in some countries or regions (Lu et al., 2022). However, inactivated vaccines face the risk of virus dispersion and strain matching at any given time, as they require the cultivation of virulent strains of viruses (Hardham et al., 2020). Moreover, the mixing of various serotype strains lead to weak cross-immunity effects and could potentially place stress on the immune system of the animals (Waters et al., 2018; Kamel et al., 2019). The efficacy of inactivated vaccines is often intricately tied to the incorporation of oil emulsion adjuvants, which enhance their protective effectiveness (Cao, 2014; Bidart et al., 2020). Nevertheless, this approach frequently results in local adverse effects, such as granulomas, fibrosis, and abscesses at the vaccination site, in addition to complications, such as maternal antibody interference (Jo et al., 2021). Although researchers have consistently devoted substantial effort to conducting alternative studies on FMD vaccines (Robinson et al., 2016), the technical challenges remain unsolved, including the potential for return of virulence and species specificity with live attenuated vaccines, subunit vaccine technology is not suitable for the serotype O FMDV, the need for multiple high-dose vaccinations with DNA vaccines, and the inadequate protective efficacy of peptide vaccines (Morgan and Moore, 1990; Rodriguez and Grubman, 2009; Kamel et al., 2019; Singh et al., 2019). Therefore, safer and more effective novel preventive immunizations are essential for the prevention and management of FMD.

The mRNA has demonstrated the ability to induce immunological activation both *in vivo* and *in vitro*, while also enhancing the translational efficiency of RNA synthesized *in vitro* (Goel et al., 2021; Kang et al., 2023). The mRNA produced through transcription reactions emerges as a promising platform for the expression of any proteins, whether *in vitro* or in cellular contexts (Rohner et al., 2022). Importantly, the mRNA vector does not carry antibiotic resistance, possesses self-adjuvant properties, does not integrate into genomic DNA within the nucleus, and exhibits strong characteristics associated with immunogenic responses (Karikó et al., 2008; Wang et al., 2021; Kobiyama and Ishii, 2022; Gote et al., 2023). With several notable outcomes achieved to date, mRNA vaccines have been utilized in the treatment and research of significant human infectious diseases, parasitic infections, tumors, and cancer (Wroblewska et al., 2015; Chaudhary et al., 2021; Barbier et al., 2022), as well as the application in animal husbandry (Furey et al., 2023; Zhao et al., 2024). The traditional development cycle for FMD vaccines is typically lengthy and resource-intensive, while research on FMD mRNA vaccines is still in its early stage.

In this study, an mRNA vaccine targeting serotype O FMD was engineered, and the immune response elicited by RNA-transfected primary splenic lymphocytes derived from guinea pigs (*in vitro*) was assessed, along with the evaluation of both immune response and vaccine efficacy in guinea pigs (*in vivo*) with immunization. The antibody titers and immune responses were also studied in pigs with vaccination. The findings revealed in this study demonstrated that the serotype O FMDV mRNA vaccine provided protection to guinea pigs against lethal doses of FMDV, while also stimulating neutralizing antibody production in pigs. This work presents an innovative perspective on developing vaccines against acute infectious diseases in livestock and further advances research in mRNA vaccine technology.

## 2 Materials and methods

### 2.1 Ethics statement

This research was performed in compliance with the Guidelines for the Care and Use of Laboratory Animals established by National Institute of Health Guide for Care and Use of Laboratory Animals. Yunnan Academy of Animal Husbandry and Veterinary Sciences approved the experimental design (agreement code: YNASVI01-2023004; 29 May 2023). The development and nourishment of these animals adhered to the stipulations of the Yunnan Provincial Regulations on Laboratory Animal Management.

### 2.2 Construction and production of serotype O FMD mRNA vaccine

Utilizing GenBank sequence data of FMDV serotype O strain (GenBank accession KY072818), the antigen sequence contained VP1(200–213)-VP1(134–161)-VP1(134–161)-3A(21–35)-3D(56–71). Detailed sequence information was provided in Supplementary material 1. A linearized plasmid DNA template was used to synthesize single-stranded mRNA through *in vitro* transcription with T7 RNA polymerase. Lipid nanoparticles (LNPs) were formulated by mixing cationic ionizable lipid (DLin-MC3-DMA), distearoyl phosphatidylcholine (DSPC), cholesterol (ethylene glycol), and PEGylated lipid (DMPE) in a molar ratio of 50:10:38.5:1.5. The LNPs were dissolved in anhydrous ethanol as the organic phase, while the capping-purified mRNA was dissolved in a sodium acetate buffer solution as the aqueous phase (Ickenstein and Garidel, 2019). Nanoparticle synthesis system from Precision Nanosystems (Canada) was employed to perform lipid nano-microfluidic encapsulation (Jiangsu Yaohai Biopharmaceutical Co., Ltd., Taizhou, China).

### 2.3 Characterization of mRNA

The specific primary rabbit anti-FMDV antibody (produced in our laboratory; Tang et al., 2021) was incubated at a dilution ratio of 1:100, as previously described (Zhao et al., 2024). Fluorescence images were captured using a fluorescence microscope (Nikon, Japan). For re-staining, a goat anti-rabbit secondary antibody (Beyotime, China) was applied at a dilution ratio of 1:500. Dynamic light scattering analysis was performed using Nano Particle Size Analyzer (Malvern, PA, United States) to quantify the particle size and polydispersity index (PDI) of mRNA-LNPs.

The mRNA vaccine was dissolved in a 0.5% solution of phosphotungstic acid dye and then observed with a JEM-1400 PLUS (JEOL, Japan) after drying.

The guinea pigs were first immunized with the mRNA vaccine, and in 24 h, samples were collected from the inoculation site and processed to generate 0.5 μm paraffin sections. After antigen retrieval, rabbit anti-FMDV antibody (Tang et al., 2021) was used as the primary antibody, and immunohistochemical analysis was performed using a hypersensitivity kit from Fuzhou Maixin (Fuzhou, China).

### 2.4 Immunization and viral challenge design

#### 2.4.1 Experimental animals

A total of 90 SPF guinea pigs (4-week old; average body weight of 200 ± 50 g) were purchased from Yunnan Luoyu (Kunming, China), and a total of 12 SPF Banna miniature inbred pigs (2-months old; average body weight of 7 ± 1.5 kg) were obtained from Yunnan Banna Miniature Pig Inbred Key Laboratory (Kunming, China). Prior to the start of the experiment, all animals were assessed for FMD antigen and antibody, showing double negative results. The experimental animals were provided with free food in the animal room of the Yunnan Provincial Research Center for Veterinary Biological Products (Baoshan, China), with feeding conducted alternately throughout 12-h day and night cycles.

#### 2.4.2 Experimental design

The 90 guinea pigs were randomly and evenly allocated into six groups: control group (PBS 200 μL with no treatment of FMDV and vaccine) and five experimental groups, treated with FMDV only (control attack) or with both FMDV and vaccine 0.2 μg, 2.0 μg, and 20 μg mRNA-LNP vaccine developed in the present study, or the commercial inactivated vaccine, the serotype O FMDV (O/Mya98/XJ/2010 + O/GX/09-7), obtained from Jinyu Baoling (Huhhot, China). The guinea pigs were immunized in the rectus femoris muscle at weeks 0 and 2. At week 6, a challenge test was conducted using 100PGID_50_ (100 times half of the guinea pigs infected) with the serotype O FMD (GenBank accession KY072818). Clinical symptoms and survival rate of guinea pigs were recorded after 1–3 weeks of continuous surveillance. The 12 pigs were randomly and evenly categorized into 4 groups: one control group (treated with PBS of 1 mL) and three experimental groups treated with either mRNA-LNP vaccine (15 μg and 30 μg) or the commercial inactivated vaccine, with the vaccine inoculation performed at the rectus femoris.

### 2.5 Elisa

The concentrations of IL-2, IFN-γ, TNF-α, and IL-1β in the supernatant of mRNA-transfected cells, guinea pig serum, or pig serum were determined using the specific kits (Shanghai Yuanye, China) according to the manufacturer’s guidelines. The serotype O FMDV liquid-phase blocking ELISA detection kit (Lanzhou Veterinary Research Institute, Chinese Academy of Agriculture Sciences, Lanzhou, China) was employed to detect the neutralizing antibodies in pigs.

### 2.6 Serum neutralizing antibody

According to the protocol previously described (Puckette et al., 2023), the serum was inactivated for 30 min at 56°C and then diluted with the maintenance solution in a dilution series from 1:8 to 1:1024. The serum was combined with 100 TCID_50_/50 μL of virus, neutralized for 1 h at 37°C, and then added with 50 μL of BHK-21 cells. The final results were determined when the virus control showed 100 TCID_50_/50 μL in a CO_2_ thermostatic incubator at 37°C for 72 h. The antibody titer was determined as the maximum dilution of the serum that could inhibit the detection of virus cytopathic effect (CPE).

### 2.7 Isolation, transfection, and flow cytometry of primary splenic lymphocytes

On days 14, 21, and 28 after immunization, guinea pigs were anesthetized with isoflurane for blood collection. Then, the guinea pigs were euthanized using excessive inhalation of isoflurane, and their spleens were aseptically collected to isolate primary splenic lymphocytes using the isolation kit (Tianjin Haoyang, China). Naked RNA was transfected into BHK-21 cells and guinea pig lymphocytes using a transfection kit (Sigma-Aldrich, Shanghai) for further cellular experiments. Cells were harvested at 6, 12 and 24 h after RNA transfection. Lymphocyte cells with CD3^+^ (Thermo Fisher, United States), CD4^+^ (Thermo Fisher, United States), and CD8^+^ (BIO-RAD, USA) were detected by flow cytometry (Becton LSR Fortessa, United States) after antibody incubation.

### 2.8 Lymphocyte proliferation and cell survival rate assay

The RNA-transfected primary guinea pig lymphocytes were stimulated with 100 μL/well of recombinant FMDV VP1 protein (Shanghai Yudo, China) for 72 h. Then, the lymphocyte proliferation rate was determined using CCK-8 kit according to the manufacturer’s instructions (APExBIO, United States).

A total of 50 μL of inactivated pig immune serum was neutralized with an equivalent volume of 100 TCID_50_/50 μL FMDV for 1 h. Subsequently, 50 μL of BHK-21 cell suspension and 100 μL of cell maintenance solution were added to the serum sample. Then, the serum was added with 10 μL of CCK-8 and incubated at a constant temperature (37°C) for 3 h. The absorbance was measured at 450 nm, using the cell blank as a control. The absorbance of the sample was determined by subtracting the absorbance of the cell blank well from that of the sample treatment well, and the inhibition rate was calculated according to the provided instructions.

### 2.9 Clinical symptoms, hematoxylin and eosin staining and immunofluorescence

The body temperature and weight of the animals were recorded during inoculation and subsequent challenge tests. Statistical tables were generated to assess the eating habits and behaviors of the animals, while adverse reactions were monitored to evaluate the safety of the vaccine for the animals.

The guinea pigs were clinically evaluated 42 d after the challenge tests, as previously described (Alves et al., 2009). Mucosal blistering or ulceration was each scored up to 6 points, while death was assigned a score of 10 points. Tissues were obtained for paraffin embedding and sectioning, and hematoxylin and eosin (H&E) staining of the sections was carried out according to the manufacturer’s instructions (Biosharp, Hefei, China).

Two pathology experts independently conducted a double-blind analysis of the tissue samples under 400 × magnification. In each treatment group, either six guinea pigs or three immunized pigs were subjected to pathogenic treatment, with 10 fields randomly selected per slide for pathological scoring. For cardiac tissue (Laster et al., 1994), the scoring parameters included interstitial edema, hemorrhage, neutrophil infiltration, and necrosis, which were evaluated based on a 4-point scale: 0 (no inflammation), 1 (mild), 2 (moderate), and 3 (severe). The combined score for these parameters was recorded as the cardiac pathology damage score. In hepatic tissue, randomly selected sections were assessed for lobular inflammation and portal vein inflammation on a 4-point scale, and for necrosis using a separate grading scale, i.e., 0 (no necrosis), 1 (<10% hepatic parenchyma), 2 (10–25% hepatic parenchyma), and 3 (˃25% hepatic parenchyma). The combined scores represented the liver pathology injury score (Siegmund et al., 2002). For splenic tissue, scoring ranged from 0 to 3 as follows: 0 (normal, unstimulated spleen with primary follicles), 1 (mild, stimulated spleen with secondary follicles), 2 (moderate, indicating some pathology), and 3 (severe, necrosis present) (Pilgrim et al., 2007). In pulmonary tissue, four parameters, including alveolar septal congestion, alveolar hemorrhage, intra-alveolar fibrin, and intra-alveolar infiltrates, were assessed using a 0–3 severity scale. The total score was calculated as previously reported (Ionescu et al., 2012). Renal tissue analysis involved scoring 100 randomly selected cortical tubules across 10 non-overlapping areas using an additive approach with a maximum score of 10 points. Parameters included tubular epithelial cell flattening (1 point), brush border loss (1 point), cell membrane bleb formation (1 or 2 points), interstitial edema (1 point), cytoplasmic vacuolization (1 point), cell necrosis (1 or 2 points), and tubular lumen obstruction (1 or 2 points) (Paller et al., 1984).

Following viral challenge, spleens from guinea pigs were fixed and processed to make paraffin-embedded sections. After antigen retrieval, the sections were subjected to immunofluorescence staining. For the detection of CD4^+^ and CD8^+^ T lymphocytes, sections were incubated overnight at 4°C with a mouse monoclonal anti-CD4^+^ T cell antibody (GB13064-2#, Servicebio, Wuhan) at a 1:500 dilution and a rabbit monoclonal anti-CD8^+^ T cell antibody (GB15068, Servicebio, Wuhan) at a 1:300 dilution. For macrophage labeling, sections were incubated overnight at 4°C with a mouse monoclonal anti-F4/80 antibody (GB12027#, Servicebio, Wuhan) at a 1:800 dilution and a rabbit polyclonal anti-CD68 antibody (GB115630#, Servicebio, Wuhan) at a 1:400 dilution. After three 5-min washes with PBS, sections were incubated for 2 h in the dark with CY3-conjugated goat anti-mouse IgG and Alexa Fluor 488-conjugated goat anti-rabbit IgG, each diluted 1:500. The slides were then mounted with an anti-fade mounting medium and examined using an immunofluorescence microscope (Nikon, Japan).

### 2.10 Western blot analysis

BHK cells of each well were treated with 10 μg of mRNA vaccine for 24 h to evaluate antigen expression after mRNA vaccination. Proteins were lysed using RIPA lysis buffer (Beyotime, China) following the protocols previously described (van de Ven et al., 2022). Equal amounts of proteins were subjected to SDS-PAGE and then electrotransferred to nitrocellulose membranes (Pall, New York, United States). The membranes were blocked and treated with designated primary antibodies, as previously described (Tang et al., 2021), and then incubated with secondary antibodies, and color development was conducted using the ECL kit (Tanon, Shanghai, China).

### 2.11 Viral load

RNA was extracted from 500 μL serum samples following the operational instructions of the RNA Extraction Kit (Yeasen Biotechnology Co., Ltd., Shanghai, China). Complementary DNA (cDNA) was prepared using the Reverse Transcription Kit (Universal Blue qPCR SYBR Green Master Mix, Yeasen Biotechnology Co., Ltd., Shanghai, China). Primers targeting the 4,377–4,501 coding region of the FMDV genome (GenBank accession KY072818) were synthesized by Sangon Biotech Co., Ltd. (Shanghai, China) and cloned into the pBM16A expression vector. The cloned sequence was verified by sequencing. A standard curve was generated by gradient dilution of the *in vitro*-synthesized RNA based on measured concentrations. Absolute quantification was performed using RT-qPCR (7,500 Real-Time PCR System, BIO-RAD, United States) with SYBR Green (Bio-Rad, USA) to evaluate viral RNA copy numbers in the samples. The upstream primer sequence was 5′-ctcaagcacgtgacatcaa-3′, and the downstream primer sequence was 5′-ctaacaaacttctcttctga-3′. A linear regression model, derived from serial dilutions of the *in vitro*-synthesized RNA standard, was used to convert cycle threshold (Ct) values into FMDV genome copy numbers (Parida et al., 2007).

### 2.12 Data analysis

GraphPad Prism 10 program was used for statistical analysis and graphical presentation. All data were presented as mean ± standard deviation (SD). Mixed effects analysis, one-way analysis of variance (ANOVA), and Tukey’s multiple comparisons were performed to evaluate the statistical significance of the differences among the groups based on *p* < 0.05 (*), *p* < 0.01 (**), *p* < 0.001 (***), and *p* < 0.0001 (****), respectively.

## 3 Results

### 3.1 Construction and expression of the FMDV serotype O mRNA vaccine

**Figure 1:**
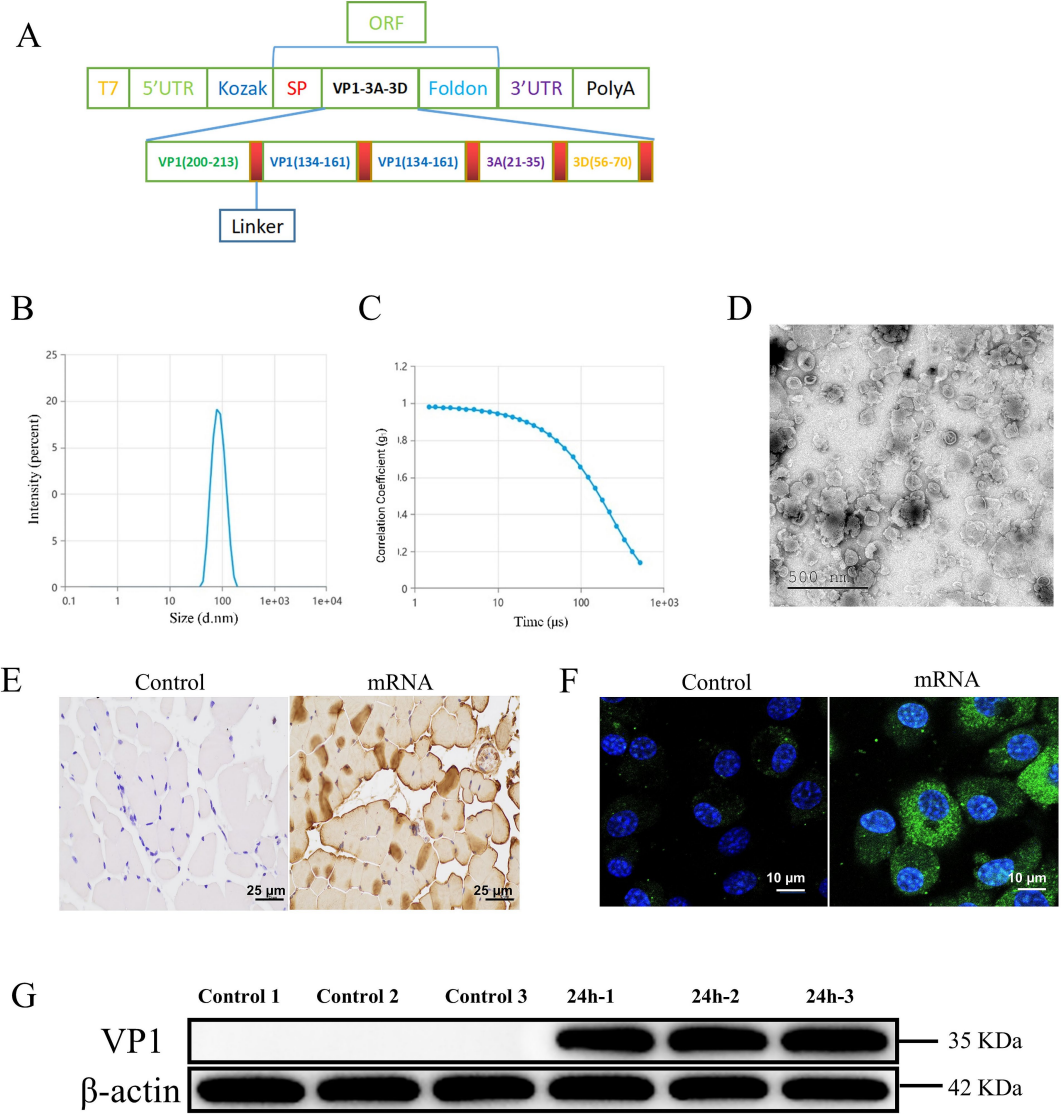
Construction and expression of serotype O FMDV mRNA vaccine. The control group was incubated with PBS. **(A)** Schematic representation of the mRNA design, containing several linear neutralizing epitopes linked by linkers. **(B)** Particle size diagram of the mRNA. **(C)** Polymer dispersion index (PDI) graph of mRNA. **(D)** Transmission electron microscopy observation of FMDV mRNA vaccine. **(E)** Immunohistochemical analysis of guinea pig rectus femoris. **(F)** Immunofluorescence analysis of FMDV mRNA transfected into BHK cells. Green denotes the VP1 protein and nucleus are indicated in blue, respectively. **(G)** Western blotting analysis of anti-FMD proteins in spleen lymphocytes from guinea pigs.

We designed an serotype O FMDV major antigen protein sequence connected by linkers. To enhance antigen expression, secretion, and the immunogenic efficacy of the mRNA vaccine, we introduced the human tissue plasminogen activator signal peptide (SP) sequence downstream of the Kozak sequence (Kou et al., 2017). Additionally, a trimeric motif from the bacteriophage T4 fibritin, known as the Foldon sequence, was inserted upstream of the 3’ UTR. This Foldon sequence is linked to antigenic epitopes via linker peptides to improve protein folding, stabilize protein conformation, and enhance the resilience of antigenic proteins under extreme conditions (Papanikolopoulou et al., 2008; Qu et al., 2022). To ensure the independent activity and biological functionality of the protein, flexible linkers were employed to optimize the spatial distance between domains, which could also extend the plasma half-life of the target protein (Trinh et al., 2004) (Figure 1A; Supplementary material 1).

The mRNA transcribed *in vitro* by T7 was encapsulated in LNPs. The transmission electron microscopic observations of mRNA-LNPs revealed the double-layered structure of the encapsulated mRNA vaccine, showing the irregularly shaped vesicle-like structures with lighter-colored borders and a darker interior core (Figure 1D). The vaccine displayed uniform dispersion, with an average particle size of 83.38 nm, a PDI of 0.06, and an encapsulation efficiency of 92.3% (Figures 1B,C; Supplementary Table 1). The observed particle size, PDI, and TEM images suggest uniformity in nanoparticle size, which is favorable for efficient cellular internalization and broad biological distribution. The findings of immunohistochemical analysis indicated that in 24 h after mRNA immunization, the muscle at the injection site of guinea pigs showed a positive expression of mRNA, whereas no expression was detected in the blank group (Figure 1E). We transfected mRNA into BHK-21 cells and examined its dispersion, no specific fluorescence was revealed in the blank group, while the group with mRNA transfection showed a strong and specific bright green fluorescence expression in the cytoplasm (Figure 1F). These results were confirmed by Western blotting analysis (Figure 1G).

### 3.2 Immunogenicity of mRNA vaccine in guinea pig spleen lymphocytes

**Figure 2:**
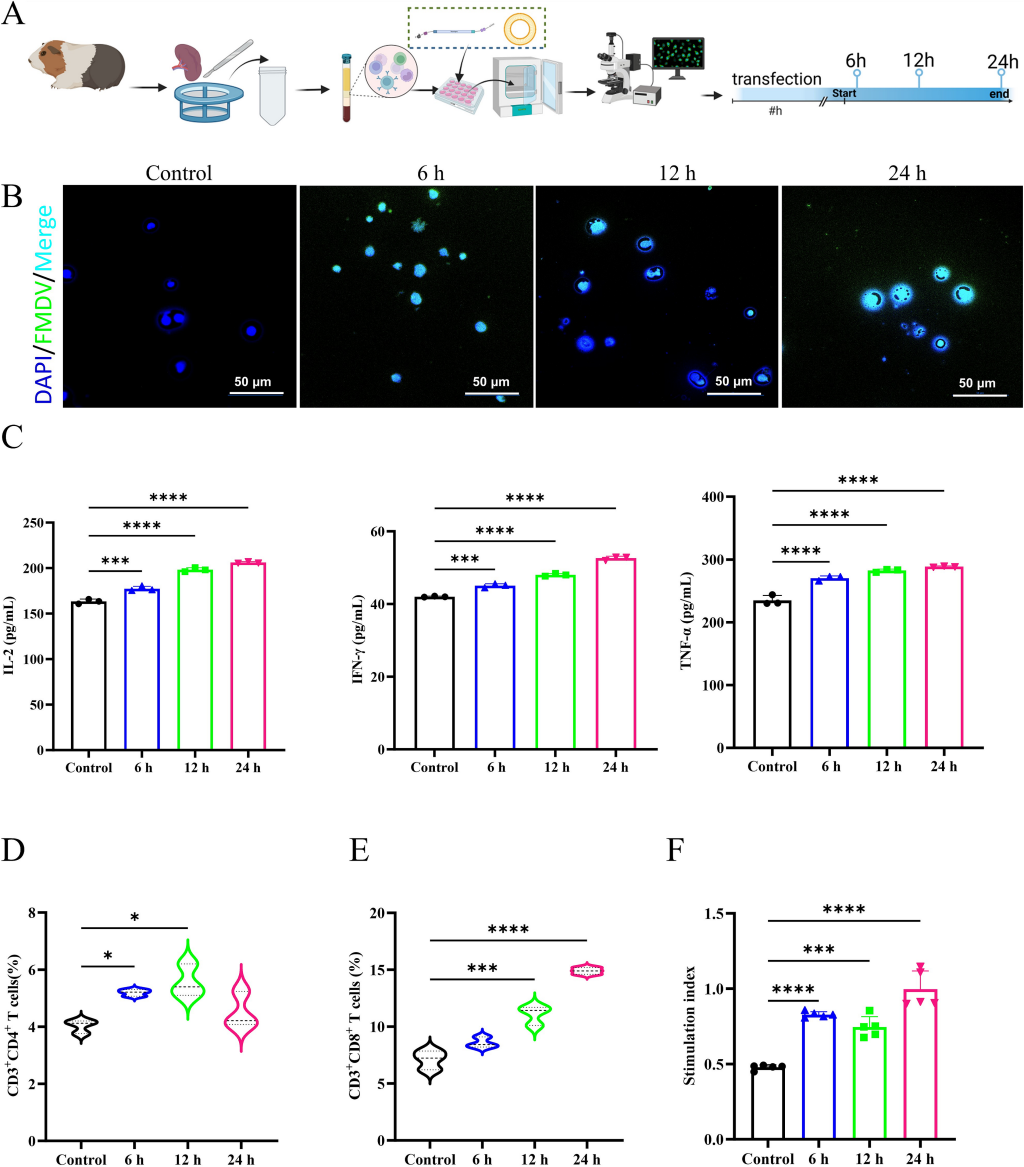
Effectiveness and immunogenicity of mRNA vaccines *in vitro* in guinea pig spleen cells. The control group was incubated with PBS. **(A)** Transfection experimental design in splenic lymphocytes. **(B)** The immunofluorescence analysis of mRNA transfection in splenic lymphatics. **(C)** Secretion of IL-2, IFN-γ, and TNF-α are presented in a horizontal sequence of images, arranged from left to right. **(D)** Evaluation of CD3^+^CD4^+^ T cells after RNA transfection of splenic lymphocytes. **(E)** Evaluation of CD3^+^CD8^+^ T cells after RNA transfection of splenic lymphocytes. **(F)** Specific lymphocyte proliferation assay.

The results of immunogenicity of mRNA vaccine showed that after mRNA transfection (Figure 2A), the control group showed no protein expression within the cytoplasm, whereas distinct green fluorescence was detected in the cytoplasm of the mRNA transfection group (Figure 2B). The results of ELISA manifested that after mRNA transfection, the concentrations of cytokines IL-2, IFN-γ, and TNF-α in the control group were significantly lower than those in the mRNA transfection group (Figure 2C), indicating the potential of mRNA-induced cellular immune responses in guinea pig splenic lymphocytes.

In addition, our results showed that mRNA could stimulate the proliferation and differentiation of CD4^+^ T and CD8^+^ T lymphocytes in the guinea pig spleen (Figures 2D,E), suggesting the potential of mRNA vaccines to activate the guinea pig’s cellular immune response. The number of CD8^+^ T lymphocytes was relatively predominant, while the number of CD4^+^ T lymphocytes declined after 12 h. The FMDV VP1 recombinant protein was used to stimulate the transfected cells, and the number of lymphocytes in the FMD mRNA transfection group was significantly higher than that of the blank control group (Figure 2F), suggesting that the mRNA could stimulate the proliferation of guinea pig lymphocytes. In summary, the mRNA constructed in this study was effective in inducing the immune response of spleen lymphocytes.

### 3.3 Immunogenicity and efficacy of FMDV mRNA vaccine in guinea pigs

**Figure 3:**
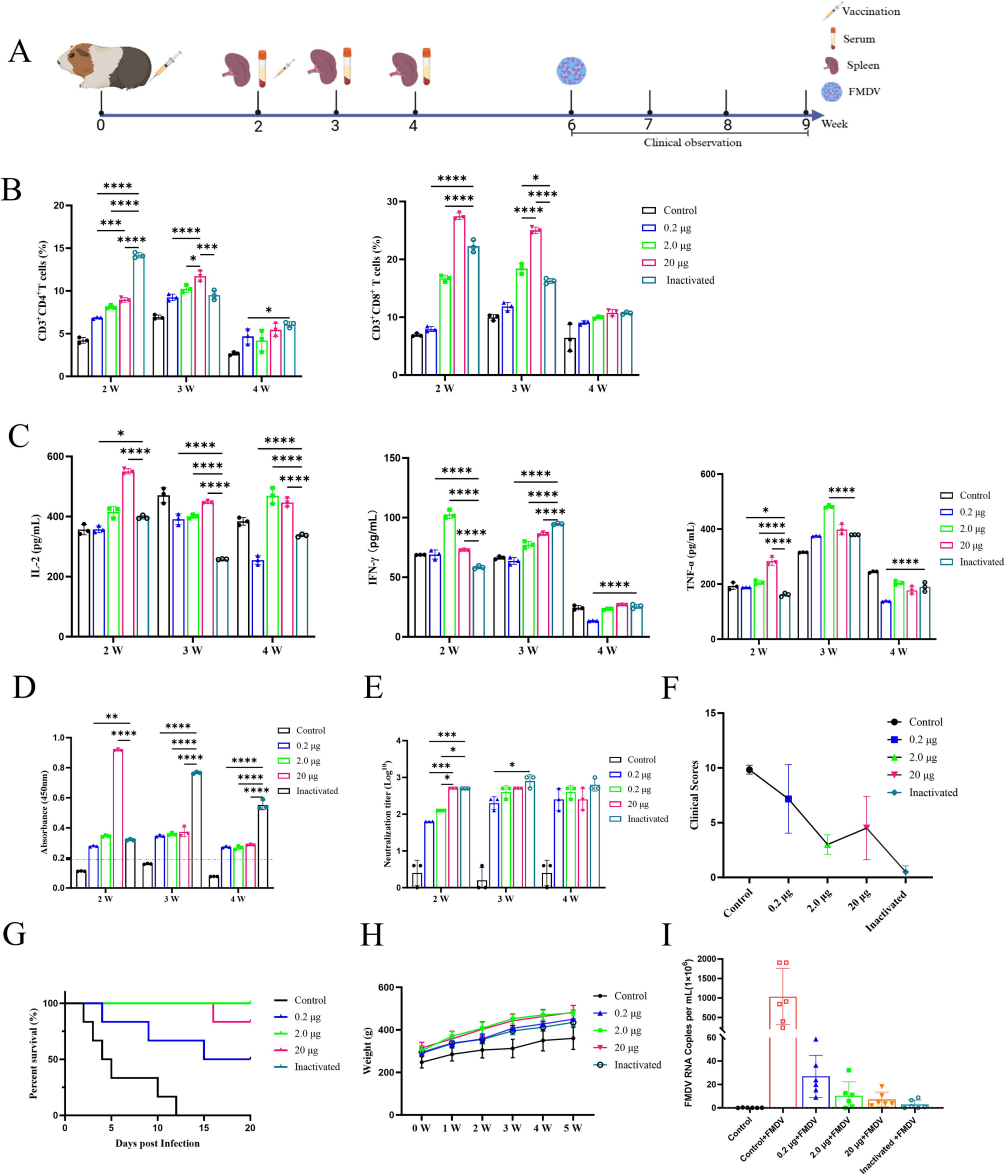
Immunogenicity and efficacy of FMDV mRNA vaccine in guinea pigs. **(A)** Experimental design. PBS as control group. **(B)** Analysis of CD4^+^ T and CD8^+^ T lymphocytes from splenic lymphocytes of guinea pigs is displayed in a row of images, arranged from left to right. **(C)** Concentrations of IL-2, IFN-γ, and TNF-α in guinea pig serum are shown in a row of pictures, from left to right. **(D)** Specific IgG antibody levels in guinea pigs. **(E)** Serum antibody levels in guinea pigs. **(F)** Clinical scores of guinea pigs after challenge tests. **(G)** Survival curves of guinea pigs after challenge test. **(H)** Body weight change curves of guinea pigs after immunization. **(I)** Viral load in guinea pigs after challenge.

The vaccination process and blood collection were depicted in Figure 3A. Flow cytometry was used to identify CD4^+^ T and CD8^+^ T cells in primary guinea pig lymphocytes following aseptic isolation and vaccination after labeling. The results demonstrated that the vaccine treatment group exhibited a higher proportion of CD4^+^ T and CD8^+^ T lymphocytes than that of the control group, while inactivated vaccines primarily elicited humoral immunity responses and displayed advantages in the early developing stages of CD4^+^ T lymphocytes in guinea pigs. The inactivated vaccines were less effective than the mRNA vaccine, which significantly elevated the number of CD8^+^ T lymphocytes in guinea pigs and triggered a more robust cellular immune response (Figure 3B). Furthermore, the results showed that the secretion of IL-2, IFN-γ, and TNF-α in guinea pig serum was elevated in the 2nd week following immunization compared to the control group. The mRNA vaccination group was the first to generate substantial levels of cytokine release, followed by the inactivated vaccine group (Figure 3C). Moreover, the results showed that the specific IgG antibody and serum-neutralizing antibody titers reached their highest levels in the mRNA vaccine group at the earliest time points. In contrast, the inactivated vaccine group exhibited a delayed peak but sustained levels for a longer duration (Figure 3D). No significant difference was observed in the results of serum neutralization antibody test between the mRNA vaccine and the inactivated vaccine of guinea pigs (Figure 3E).

**Figure 4:**
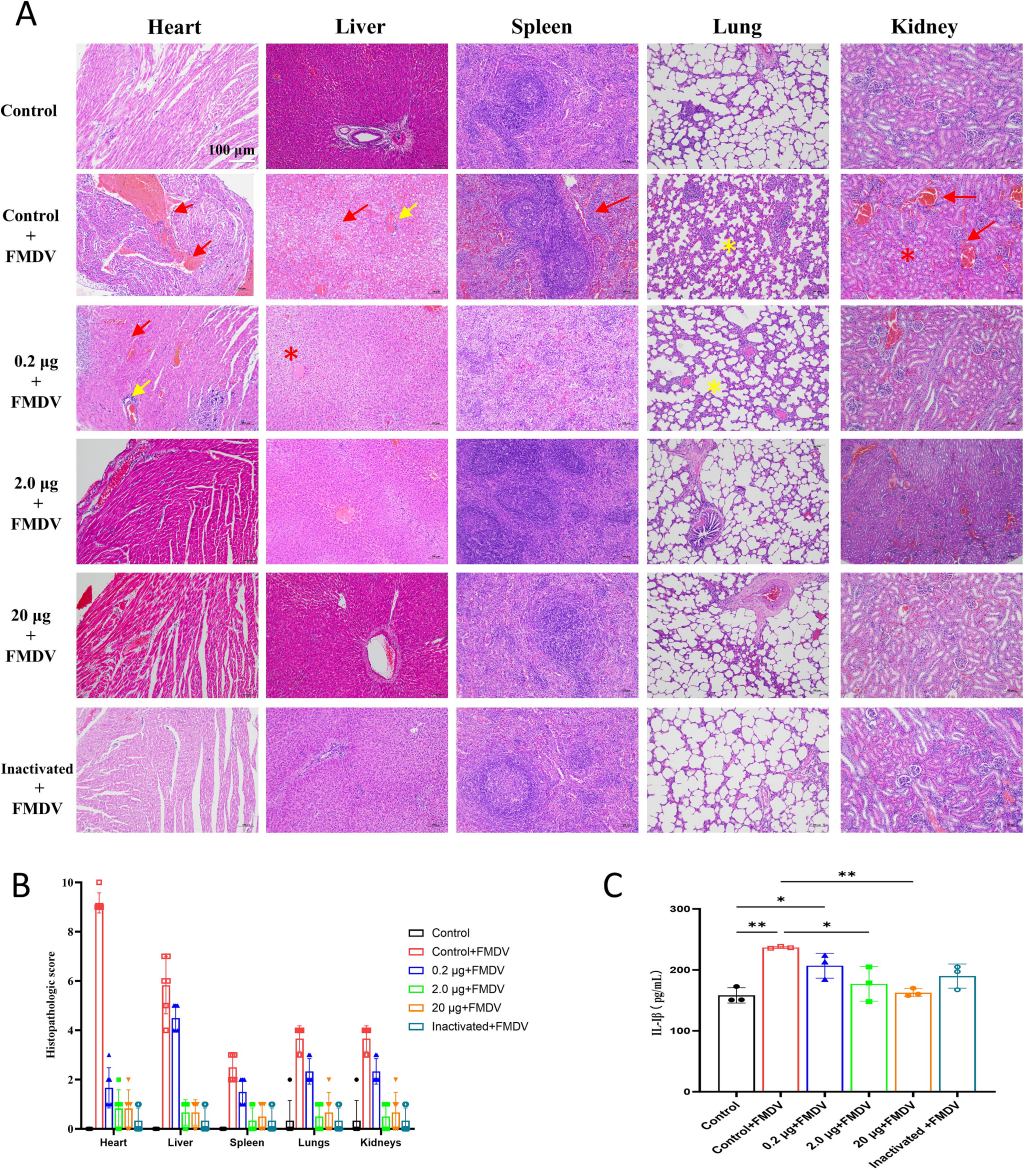
**(A)** H&E staining of guinea pig organs after challenge tests. Damaged areas are indicated by green arrows. Scale bar of 100 μm is applied to all images (40 × magnification). Bleeding and blood stasis are indicated by red arrows, necrosis by red stars, lymphocyte infiltration by yellow arrows, and alveolar wall widening by yellow stars. **(B)** Pathological scoring chart of organs in guinea pigs after challenge. **(C)** Concentrations of 1 L-1β in guinea pigs after challenge.

In addition, the guinea pig blank challenge group showed the earliest onset of infection symptoms and the highest clinical score, with all animals perishing 12 d later (Figure 3F). The inactivated vaccine group demonstrated a 100% protection rate (i.e., all 6 guinea pigs survived), the mildest symptoms, and the lowest clinical score. The group treated with 2.0 μg mRNA vaccine achieved a clinical score of 3.0, demonstrating high resilience to a challenge at 100 times the lethal dose of GPID_50_, and achieving a protective level comparable to that of the inactivated vaccine. In the group that received 20 μg mRNA, one animal died on day 16 and another experienced a brief period of mental impairment before recovering, resulting in an 83.3% survival rate (5 out of 6 animals survived) and a clinical grade of 4.5. The group treated with 0.2 μg mRNA vaccine suffered a low protection rate of 50% (3 out of 6 animals survived) with three deaths detected on days 4, 9, and 15 (Figures 3F,G). In addition, viral load analysis following challenge in guinea pigs revealed that the mRNA vaccine group significantly reduced FMDV copy numbers (Figure 3I). Furthermore, analysis of serum inflammatory cytokine IL-1β levels indicated that the vaccine group exhibited reduced expression of this inflammatory mediator, whereas the blank challenge group showed a marked increase in IL-1β levels (Figure 4C).

Statistical analysis revealed that vaccinated guinea pigs had higher body weights than controls after immunization (Figure 3H), indicating that the vaccine showed no impact on body weight gain. Concurrently, H&E staining of the heart, liver, spleen, lung, and kidney of the blank challenge group revealed widespread pathological damages, such as heart, liver, spleen, kidney focal or large area bleeding and blood stasis, liver lymphocyte infiltration, alveolar wall widening. The 0.2 μg group showed localized cardiac ecchymosis, lymphocyte infiltration, hepatocyte necrosis, and expansion of alveolar wall (Figure 4A). The vaccination group showed varying degrees of decreased or even prevented viral invasion. To accurately assess the pathological damage to guinea pig organs following viral exposure, statistical analysis was performed based on a pathological scoring system. Results indicated that the Control+FMDV group exhibited the most severe pathological damage, followed by the 0.2 μg mRNA group, which had significantly higher organ pathology scores compared to the control group and other vaccine-treated groups. No significant differences were detected among the 2.0 μg mRNA group, the 20 μg mRNA group, and the inactivated vaccine group, as well as between these aforementioned groups and the control group, consistent with clinical scoring results (Figure 4B). In summary, it was conclude that the 2.0 μg mRNA and inactivated vaccine groups showed the highest level of efficacy, followed by the 20 μg mRNA group, with the lowest level of efficacy detected in the 0.2 μg mRNA group. Additionally, under immunofluorescence microscopy, we observed the activation and elevated expression of CD4^+^ T and CD8^+^ T lymphocytes, as well as macrophages, with effects surpassing those observed in the control group and the inactivated vaccine group (Supplementary Figure 2). In summary, inoculating guinea pigs with mRNA vaccine induced significant cellular and humoral immunity and was clinically safe, with no adverse effects detected, providing protection against a fatal dose of the FMDV.

### 3.4 Immunogenicity and effectiveness of mRNA vaccination in pig

**Figure 5:**
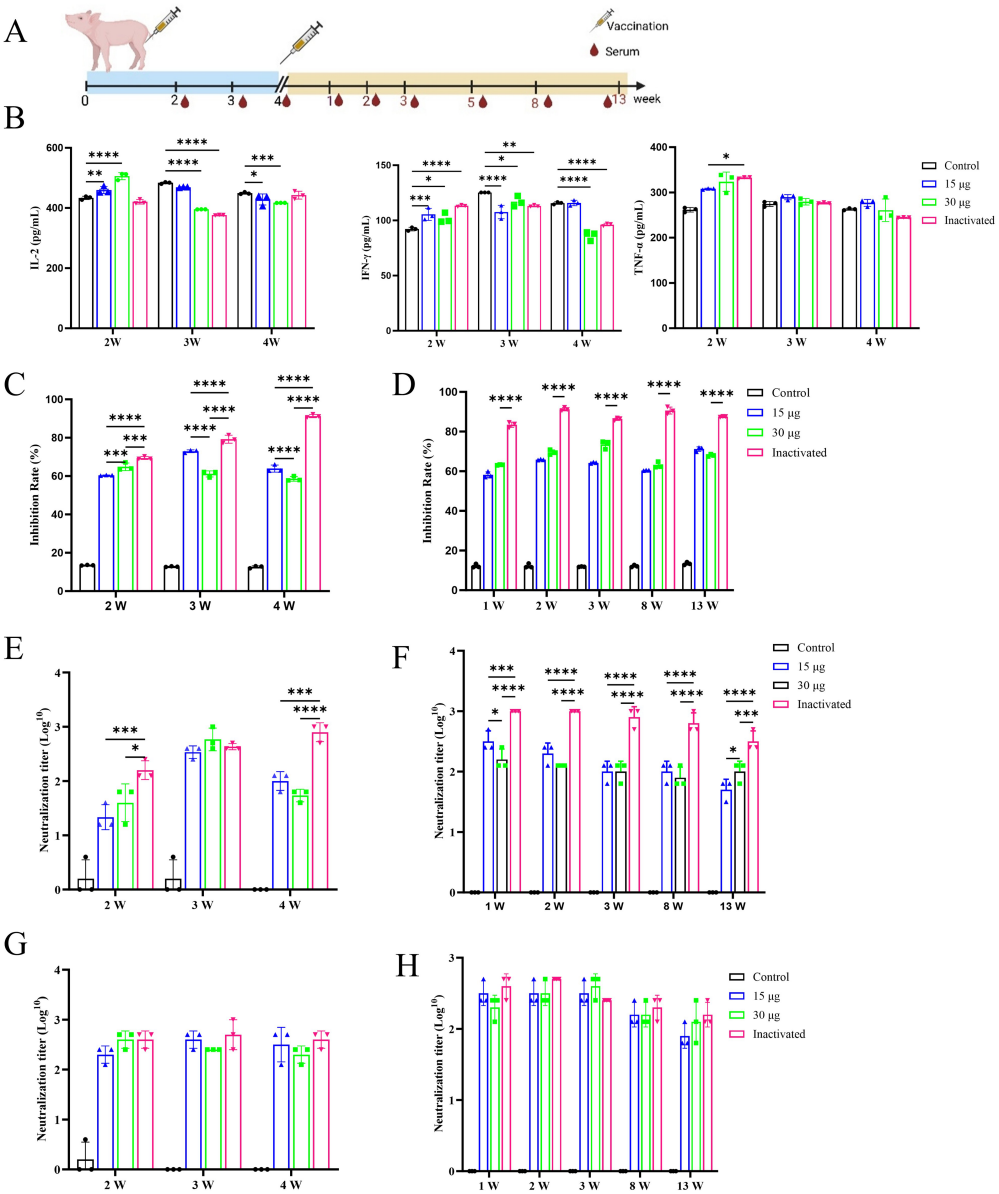
Immunogenicity and effectiveness of mRNA vaccinations in pig. All images are displayed in a single row, arranged from left to right. PBS as control group. **(A)** Experimental design of vaccination. Piglets of 2-month-old are immunized on days 1 and 28. The serum samples are collected 2, 3, and 4 weeks after the first immunization and 1, 2, 3, 5, 8, and 13 weeks after the second immunization. **(B)** The serum concentrations of IL-2, IFN-γ, and TNF-α in pigs after the first immunization. **(C)** The rate of viral inhibition of pig initial immunization. **(D)** The rate of viral inhibition in pig after immunological boosting. **(E)** The liquid-phase blocking antibody titer of pigs after first immunization. **(F)** The liquid-phase blocking antibody titer of pigs after immunological enhancement. **(G)** The serum-neutralizing antibody concentration in pigs after initial immunization. **(H)** The serum-neutralizing antibodies in pigs after immunological boosting.

The immunogenicity and efficacy of the mRNA vaccine were evaluated following the vaccination protocol and blood collection schedule depicted in Figure 5A. The early stage of vaccination induced a cellular immune response in pigs, as demonstrated by the serum concentrations of three types of cytokines (IL-2, IFN-γ, and TNF-α) in the vaccine-treated group, which exhibited an increasing trend at week 2 after the first immunization and then reverted to the similar levels to that of the control group at week 4 (Figure 5B). Subsequently, the results showed that the pig immune serum could effectively improve the survival rate of BHK-21 cells after incubation. The mRNA vaccine group also maintained a favorable cell growth state, achieving viral inhibition rates of 58.19–72.95% over a period of 4 months, which were significantly higher than those of the control blank group (Figure 5C).

**Figure 6:**
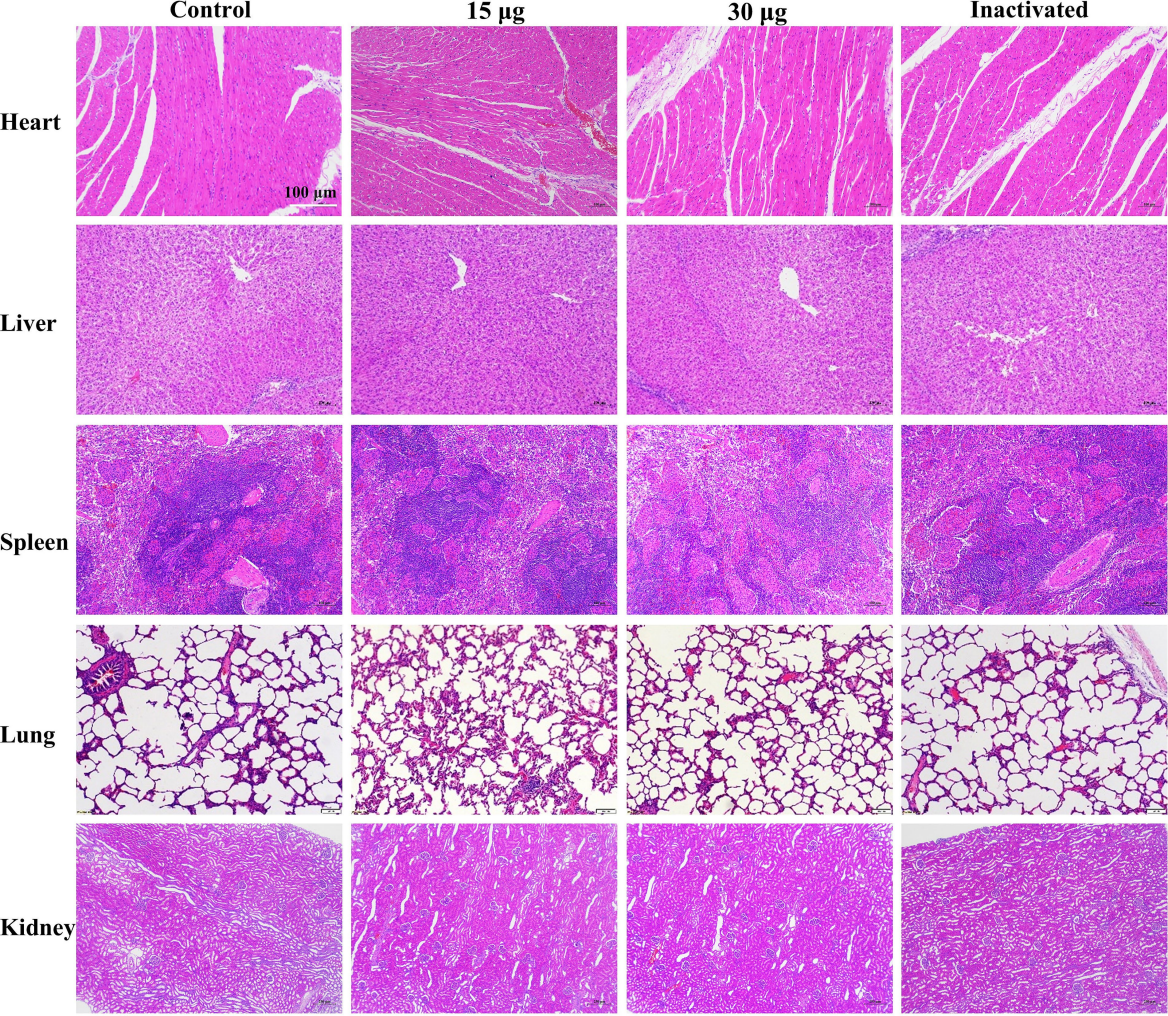
H&E staining of pig organs after vaccination. Scale bar of 100 μm is applied to all images (40 × magnification).

During the 3rd week after the initial immunization, no distinguishable difference in the level of antibody titer was detected between the groups treated with mRNA vaccine and inactivated vaccine, whereas at other times, the titer was lower than that of the inactivated vaccine group (Figure 5D). No significant difference was observed in the level of serum-neutralizing antibody titers between the groups treated with inactivated vaccine and mRNA vaccine (Figure 5E). Through clinical observation of vaccinated pigs, pathologists conducted a blinded histopathological assessment of organ samples 17 weeks post-immunization. No significant differences were detected in histopathological scores between the vaccine-treated and control groups, and no pathological damage was observed under microscopic examination. Consequently, histopathological scoring data were not presented for post-immunization pigs. These findings indicated that the mRNA-LNP vaccine was safe and reliable, with no adverse pathological effects observed (Figure 6). Furthermore, under immunofluorescence microscopy, the mRNA vaccine group similarly exhibited higher expression of CD4^+^ and CD8^+^ T lymphocytes and macrophages compared to the control group and the inactivated vaccine group (Supplementary Figure 3). In summary, the inoculation of the serotype O FMD mRNA vaccine in pigs elicited cellular and humoral immunity and was clinically safe, with no adverse effects detected.

## 4 Discussion

Although FMD vaccinations have been essential in managing and eradicating outbreaks of FMD in certain areas, this disease continues to pose a threat to animals in those regions, and the authorized vaccines fail to effectively mitigate the risk of future outbreaks (Jamal and Belsham, 2013; de Los Santos et al., 2018). Consequently, developing novel protective vaccines for FMD is the most effective strategy for disease prevention. In comparison to other vaccinations, mRNA vaccines are simpler and faster to produce, characterized by a short manufacturing and development timeline, along with a favorable safety profile (Chaudhary et al., 2021; Park et al., 2021; Barbier et al., 2022). By encoding a portion of the viral antigen genes, the mRNA vaccine omits other sequences, such as the plasmid backbone and viral promoters, exhibiting no potential for genome integration or cell transformation, avoids insertion mutagenesis, such as DNA vaccines, and reduces the risk of potential infection (Rahman et al., 2021). Additionally, mRNA vaccine can express proteins without crossing the nuclear barrier, allowing immediate expression (Linares-Fernández et al., 2020). Studies have shown improved regulation of antigen exposure and a reduced likelihood of tolerance induction (Minervina et al., 2022). Furthermore, mRNA vaccines allow for repeated administration and can encode multiple genes of interest (Pardi et al., 2018). Even low doses of mRNA can still result in higher and more sustained protein expression, therefore, it is crucial to reduce the required dosage and make it an advantageous approach for biotherapy (Sahin et al., 2014). It is well known that mRNA vaccines have several advantages over traditional live viral, protein, and polypeptide vaccines (Arevalo et al., 2022; Qin et al., 2022), in particular, they have a higher antigen load, and can trigger a strong and sustained immune protection effect, induce the cellular and humoral immunity responses in the body, and avoid biosafety risks, such as the culture of numerous virulent strains associated with inactivated vaccines (Aljabali et al., 2023). Additionally, attenuated live may exhibit virulence reversion or interfere with the initial immune response (Zhou et al., 2016; Kenubih, 2021). However, mRNA vaccines typically induce immunogenicity by utilizing host cell translation platform to enhance the production of encoded proteins, leading to strong cellular and humoral adaptive responses (Abdelaziz et al., 2024). Despite these benefits, there have been no reports of mRNA vaccination against FMD. Herein, we assessed the immunoprotective efficacy of the FMD mRNA vaccine via *in vitro* and *in vivo* animal studies. The results confirmed that the mRNA vaccine could protect guinea pigs against FMDV infection and generate neutralizing antibody titers comparable to those of the inactivated vaccines. These findings revealed in the clinical trials provide a foundation for the further development and application of the FMD mRNA vaccine.

Increasing research has focused on cross-immunity in FMD with the aim of developing a universal FMD vaccine capable of simultaneously protecting against several subtypes or serotypes (Shao et al., 2024). The mRNA vaccine provides a platform for exploring multi-functional FMD vaccines, as it can simultaneously express multiple target genes, facilitating the development of broad-spectrum vaccines and providing researchers with innovative ideas and approaches for developing universal FMD vaccines (Wu et al., 2022; Xiong et al., 2023). The main antigen of FMDV, known as VP1, is the principal surface protein. It is responsible for inducing the host CD8^+^ T cell response, neutralizing antibody response, and cross-protection against other FMDVs (Li Q. et al., 2023). To date, VP1 protein and its antigenic determinants have become the primary focus of research in the development of new vaccines (Yang et al., 2020). Studies have shown that the RGD (Arg-Gly-Asp) motif binds to VP1 and interacts with integrin receptors to trigger viral infection and transmit internalization signal into cells (Cubillos et al., 2008). We integrated construction strategies for the most immunogenic regional epitopes, B cell epitopes at positions 200–213 and 134–161 of amino acid residues, together with T cell epitopes 3A (amino acid residues from 21 to 35) and 3D (amino acid residues at position from 56 to 71), can elicit both cellular and humoral immunity, enhancing immunological response to FMD in animals (DiMarchi et al., 1986; Joyappa et al., 2009; Fernandez-Sainz et al., 2019). In our study, these antigenic epitopes were selected for the development of the mRNA vaccine. The findings revealed preliminary efficacy of the mRNA vaccine, suggesting its potential to serve as a universal vaccination. These results were consistent with those previously reported. For example, Jo et al. (2021) combined the active structural domain of heat shock protein 70 (HSP70) with the FMDV universal T-cell epitope 3A, the B-cell epitopes of FMDV types O and A, and the VP1 region of FMDV, resulting in the synthesis of a recombinant protein (rpHSP70-AD), which elicited both cellular and humoral immunoprotection against FMDV types O and A. Defaus et al. (2020) developed a dendritic macromolecule-coupled peptide vaccine by combining a multi-antigenic peptide epitope of FMDV via a thiol-maleimide linkage and a copper(I)-catalyzed azide-alkyne 1,3-cycloaddition (CuAAC) cycloaddition reaction, showing that a low dose of the peptide vaccine induced a long-term protective immunity in pigs. Forner et al. (2021) conducted analogous research on the development of a novel second-generation peptide vaccine by generating a conformation-dependent dendritic structure composed of a polymer of FMD B- and T-cell epitopes to address the limited half-life of linear peptide vaccines and enhance the broad-spectrum immunity. Chathuranga et al. (2022) identified a soluble multi-epitope antigen recombinant protein encompassing the antigenic sites of the three topological types (amino acid residues VP1 132–162 and VP1 192–212) of type O and type A FMDV. Following emulsification with adjuvants, this protein could provide complete protection to pigs against homologous residues and 75% protection against heterologous viral attacks. In summary, out study of FMDV mRNA vaccine shows significant potential to facilitate the development of a multifunctional, broad-spectrum vaccination exhibiting cross-immunity effects.

CD4^+^ T cells promote the production of protective antibodies by driving B cell differentiation into memory B cells and long-lived plasma cells, while CD8^+^ T cells eliminate infected cells through cytotoxic activity, contributing to the establishment of long-term immune memory. This synergistic interaction between T and B cells provides a critical mechanism for establishing durable immune protection, laying a scientific foundation for optimizing FMD vaccine design (Guzman et al., 2010; Carr et al., 2013). Post-mRNA immunization, the proportions of CD4^+^ and CD8^+^ T lymphocytes in guinea pig serum were significantly higher than those in the control group (Figure 3B). Similarly, immunofluorescence microscopy revealed elevated expression of CD4^+^ and CD8^+^ T lymphocytes compared to the control group (Supplementary Figures 2A, 3A). IL-1β, a key proinflammatory cytokine in FMD, serves as both an indicator of innate immune activation following FMDV infection and a driver of pathological inflammation. Its elevated expression not only acts as a biomarker for assessing FMD severity, but also holds potential as a therapeutic target (Zhi et al., 2020; Choudhury et al., 2021). In this study, we observed an increase in IL-1β expression in guinea pigs post-challenge, whereas the mRNA-immunized group significantly suppressed its expression (Figure 4C), underscoring the potential application value of this vaccine. Macrophages, constituting 1–5% of cells across all organs, function as sentinel cells by monitoring infection and abnormalities through phagocytosis and scavenger receptor pathways. They maintain homeostasis and clear pathogens and cellular debris (Cox et al., 2021), and their remarkable plasticity renders them essential in development, tissue repair, and immunity. In infectious diseases, macrophages form the first line of defense by engulfing pathogens and releasing proinflammatory cytokines, such as TNF-α (Wynn et al., 2013; Lazarov et al., 2023). This study (Supplementary Figures 2B, 3B) aligns with the findings of Zhi et al. (2018), suggesting that macrophages play a pivotal role in recognizing FMDV and initiating antiviral responses, thereby contributing significantly to controlling FMDV infection and modulating immune responses. Collectively, our vaccine exhibits pronounced immunogenicity, which serves as the foundation for its protective efficacy.

The humoral immune response is a crucial element of vaccinations, and strong association has been reported between neutralizing antibodies and the protective efficacy of vaccines (Mayr et al., 2001; Mahapatra and Parida, 2018). Our study showed that the mRNA vaccine demonstrated the same immune protective effect on guinea pigs as that of the inactivated vaccine (Figure 3G). Additionally, no significant difference was detected in the serum neutralizing antibody titer between guinea pigs (Figure 3E) and pigs (Figure 5E). Notably, the guinea pig challenge experiment demonstrated that the mRNA vaccine effectively mitigated viral damage to organs and significantly reduced viral load, achieving an immune protective efficacy comparable to that of the inactivated vaccine group (Figures 4B,C). Furthermore, the results of our study confirmed that the mRNA vaccine induced cellular and humoral immunity in the animals more quickly and earlier than the inactivated vaccine. The specific IgG antibody of guinea pigs (Figure 3D) and the mRNA vaccine in pigs (Figure 5D) reached the peak of antibody earlier of initial immunization, compared with the traditional inactivated vaccine, and the mRNA vaccine induced the secretion of cytokines at higher levels than the inactivated vaccine at the initial stage of immunization (Figures 3C, 5B). These findings were consistent with those of the SARS-CoV-2 mRNA vaccine (Karbiener et al., 2022; Luan et al., 2023). This implies that the development of the FMD mRNA vaccine could offer methods and concepts for creating emergency vaccinations. However, it has also been noted that the maintenance effect of mRNA vaccine antibodies was somewhat inferior to that of traditional inactivated vaccines (Figure 5D), and this was because that a whole virus-coated plate was used in ELISA, allowing for the retention of all antigen epitopes present in the infected particles. In contrast, inactivated vaccines combine multiple antigens to address the issue of viral antigen diversity, leading to inherently high detection results. However, this immunization strategy is not necessarily the most effective (Kenubih, 2021). Simultaneously, there is potential for further improvement and enhancement of mRNA vaccines, including optimizing mRNA codons, modifying nucleoside, refining delivery pathways, improving cryopreservation processes, and implementing other optimization strategies to further enhance the protective effect of the vaccines (Crommelin et al., 2021; Kim et al., 2022; Oude Blenke et al., 2023; Wang et al., 2023). However, the development, purification, and detection of mRNA vaccines remain heavily reliant on expensive equipment and consumables. Moreover, the stringent cold chain requirements further escalate production costs (Rosa et al., 2021; Schoenmaker et al., 2021; Uddin and Roni, 2021). These factors contribute to the high cost of mRNA vaccines, limiting their widespread use and promotion. It is also worth noting that only a small number of animals were used in this study, and they were housed exclusively in laboratory conditions. No extensive field trials or challenge tests were conducted on the target animals under real-world conditions. Moving forward, our research will focus on optimizing the expression of the mRNA vaccine antigen, modifying nucleotide sequences to enhance stability and antigen expression, conducting challenge protection experiments on FMD target animals, and performing field trials to evaluate antibody levels. These efforts aim to confirm the vaccine’s immune protective efficacy.

Our results showed that the antibody titers were maintained at a higher level following the immunization enhancement. One week after immunization enhancement, the cytokine concentrations in the inactivated vaccine group were lower than those of the mRNA vaccine group. These results indicated that the inactivated vaccine primarily targeted humoral immunity rather than cellular immunity, and this defense mechanism was inadequate (Jo et al., 2021). Due to their ability to simultaneously elicit both humoral and cellular immunity in animals, mRNA vaccines provide advantages in the early stages of animal immune development. Additionally, after immunization, the serum concentrations of IL-2, IFN-γ, and TNF-α were increased compared to the control group, but later returned to levels similar to those of the control group (Figures 3C, 5B; Supplementary Figure 1), suggesting that the vaccine caused no excessive immune activation or an inflammatory storm in the guinea pigs and pigs. As observed by the body weight of the immunized guinea pigs (Figure 3H) and H&E staining (Figure 6), clinical evaluations (Figure 3F) following simultaneous vaccination demonstrated that the mRNA vaccine provided a safe and effective immune response, which was consistent with the outcomes of COVID-19 mRNA vaccine (Polack et al., 2020; Baden et al., 2021; Baden et al., 2024). Based on the above findings, mRNA vaccines can serve as a significant complement to the existing vaccine system or as an alternative vaccination strategy. Previous studies have demonstrated that mRNA vaccines can enhance the immunogenicity of inactivated vaccines, improve antigen expression, and elevate the level of immune protection (Wang et al., 2022; Zuo et al., 2022). Therefore, it is useful and practicable to combine the mRNA vaccine with the available inactivated vaccine.

In conclusion, the serotype O FMD mRNA vaccine developed in our study showed expression in the cytoplasm and at the site of vaccine inoculation, successfully eliciting cellular and humoral protection in both guinea pigs and pigs. The levels of neutralizing antibody titers from mRNA vaccines were constantly maintained for up to 4 months after vaccination, showing no significant difference compared to those of inactivated vaccines. Furthermore, this vaccination effectively protected guinea pigs against lethal levels of viral infection, serving as a promising foundation for developing preventative vaccinations against FMDV.

## Data Availability

The raw data and figures form the study have been archived in figshare (10.6084/m9.figshare.27753828).
