# Supplementary material for: Preliminary evaluation of a novel serotype O foot-and-mouth disease mRNA vaccine
Source: Front Microbiol. 2025 Apr 28;16:1503191. doi: 10.3389/fmicb.2025.1503191 (PMC12067417; doi:10.3389/fmicb.2025.1503191)
Supplement: Supplementary file 1 [file Data_Sheet_1.docx]

Supplementary Materials

# Sequence information for mRNA vaccines

5'-

TAATACGACTCACTATAGGGAATAAACTAGTATTCTTCTGGTCCCCACAGACTCAGAGAGAACCCGCCACCGCCACCATGGATGCAATGAAGAGAGGGCTCTGCTGTGTGCTGCTGCTGTGTGGAGCAGTCTTCGTTTCGCCCAGCCAGGAAATCCATGCCCGATTCAGAAGAAGACATAAGCAGAAGATTGTGGCACCCGCAAAACAGCTTTTGGGCGGAGGAGGCAGCGGCGGAGGAGGCAGCTGCAGGTACAGCAACAGCAACGTGAGCAAATTGAGAGGTGATCTCCAAGTGTTGGCTCAGAAGGCGGCGAGACCGCTGCCTACCGGCGGAGGAGGCAGCGGCGGAGGAGGCAGCTGCAGGTACAGCAACAGCAACGTGAGCAAATTGAGAGGTGATCTCCAAGTGTTGGCTCAGAAGGCGGCGAGACCGCTGCCTACCGGCGGAGGAGGCAGCGGCGGAGGAGGCAGCGCAGCAATTGAATTCTTTGAGGGGATGGTCCACGACTCCATTAAAGGCGGAGGAGGCAGCGGCGGAGGAGGCAGCATCTTCTCCAAACACAAAGGAGACACAAAGATGTCTGAAGAGGACGGCGGAGGAGGCAGCGGCGGAGGAGGCAGCGGAAGCGGCTACATCCCAGAAGCCCCTAGAGACGGACAGGCTTACGTGCGAAAAGACGGCGAGTGGGTGCTGCTGAGCACATTCCTGGGAAGGAGC**TAATAG**GCTGGAGCCTCGGTGGCCTAGCTTCTTGCCCCTTGGGCCTCCCCCCAGCCCCTCCTCCCCTTCCTGCACCCGTACCCCCGTGGTCTTTGAATAAAGTCTGAGTGGGCGGCAAAAAAAAAAAAAAAAAAAAAAAAAAAAAAAAAAAAAAAAAAAAAAAAAAAAAAAAAAAAAAAAAAAAAAAAAAAAAAAAAAAAAAAAAAAAAAAAAAAAAAAAAAAAAAAAAAAAAAAA-3'

# Supplementary Figures and Tables


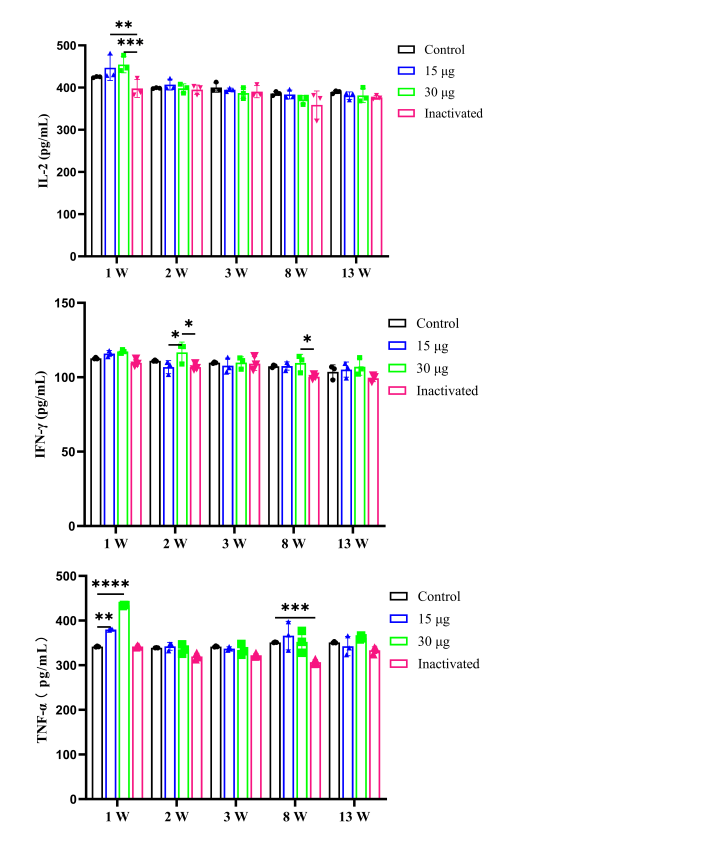


**Supplementary Figure 1.** Serum concentrations of IL-2, IFN-γ, and TNF-α in pigs with immune enhancement.

**Supplementary Table 1.** Quality control tests for the mRNA vaccine performed by Jiangsu Yaohai Biopharmaceutical Co., Ltd. (Taizhou, China).

| **Test item** | **Standard of detection** | **Actual detection** |
| --- | --- | --- |
| Particle size (nm) | < 100 | 83.38 |
| Polydispersity index (PDI) | < 0.1 | 0.06 |
| Zeta Potential (mV) | Neutral | –2.055 |
| Encapsulation efficiency | > 90% | 92.3% |
| Conclusion of quality control | Qualified | |


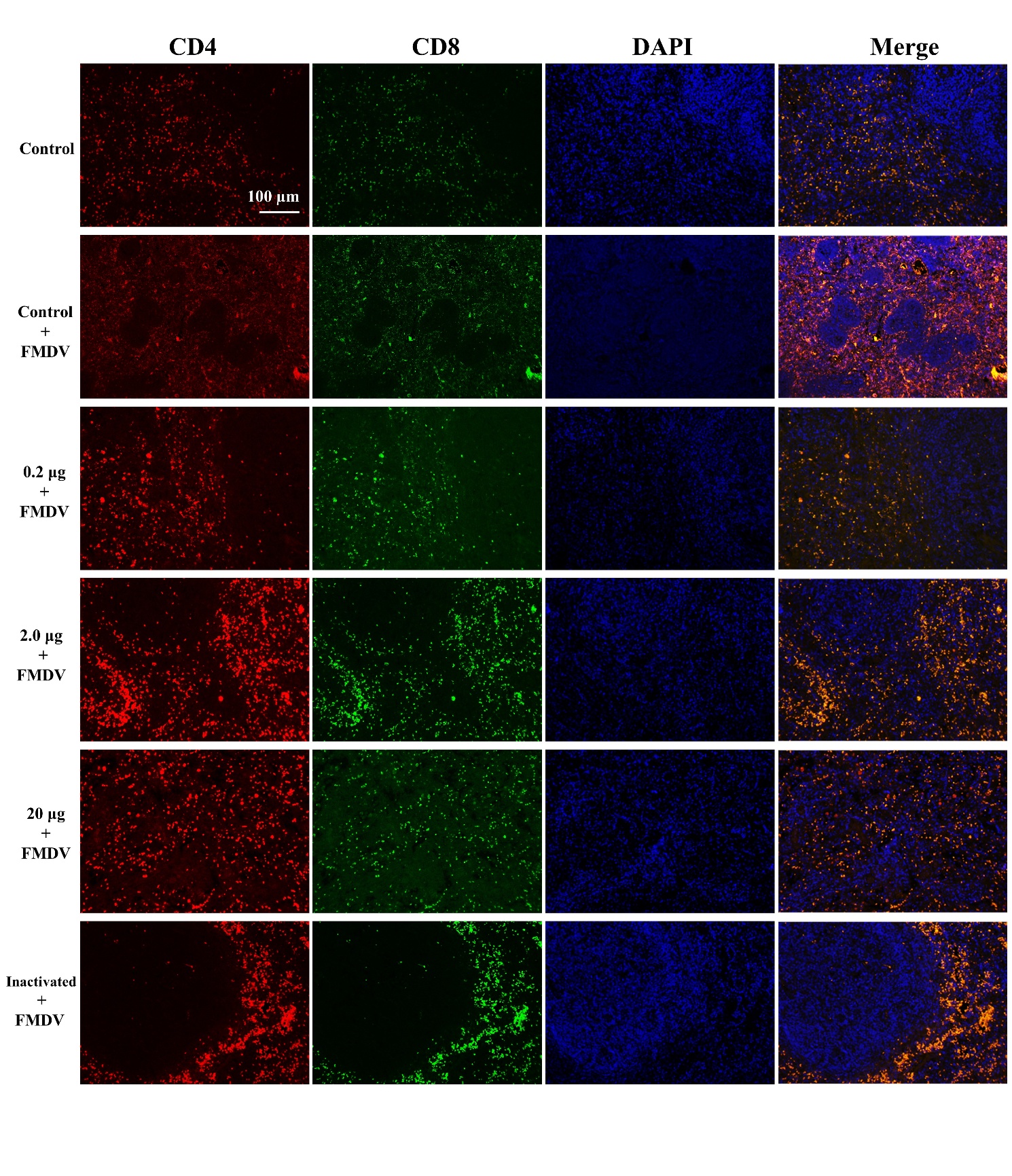


A


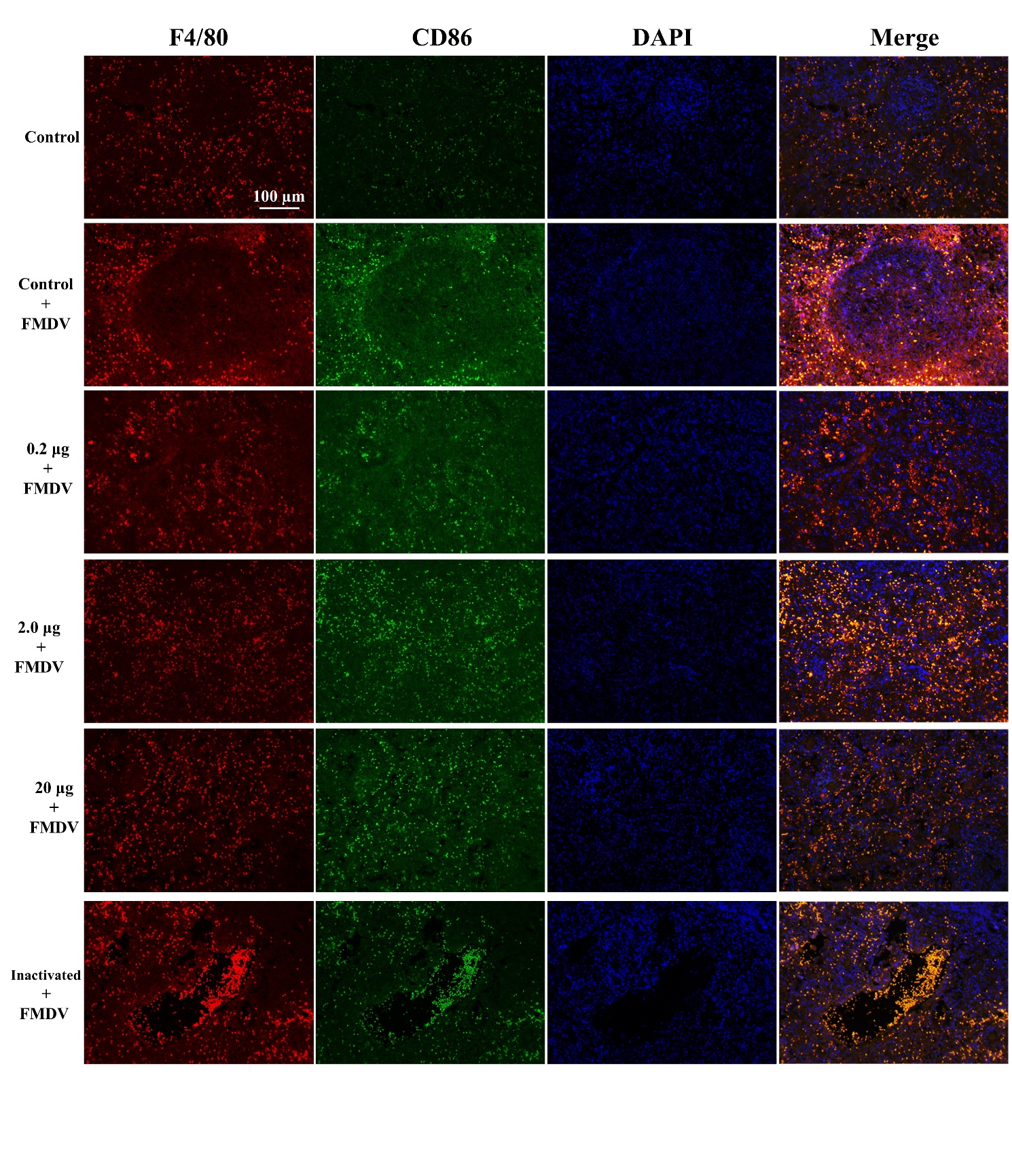


B

**Supplementary Figure 3**. Immunofluorescence of CD4^+^ and CD8^+^ T lymphocytes and macrophages in guinea pig spleen. (A). CD4^+^ and CD8^+^ T lymphocytes in guinea pig spleen. (B). Macrophages in guinea pig spleen. Scale bar of 100 μm is applied to all images (40× magnification).


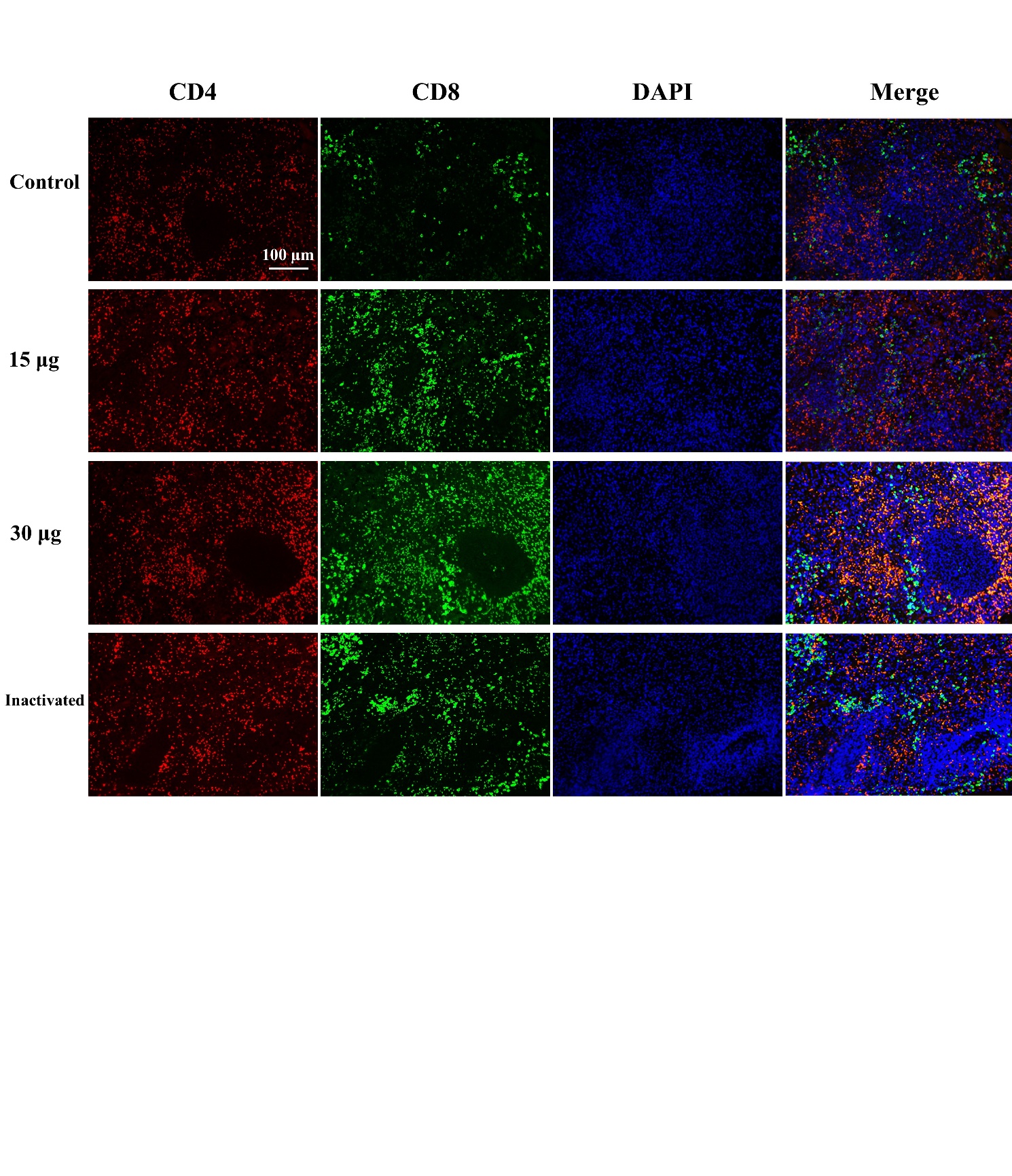


B

**Supplementary Figure 4**. Immunofluorescence of CD4^+^ and CD8^+^ T lymphocytes and macrophages in porcine spleen. (A). CD4^+^ and CD8^+^ T Lymphocytes in porcine spleen. (B). Macrophages in porcine. Scale bar of 100 μm is applied to all images (40× magnification).
